# Social Norms and Preventive Behaviors in Japan and Germany During the COVID-19 Pandemic

**DOI:** 10.3389/fpubh.2022.842177

**Published:** 2022-04-01

**Authors:** Christoph Schmidt-Petri, Carsten Schröder, Toshihiro Okubo, Daniel Graeber, Thomas Rieger

**Affiliations:** ^1^Department of Philosophy, Karlsruhe Institute of Technology, Karlsruhe, Germany; ^2^Socio-Economic Panel, DIW Berlin (German Institute for Economic Research), Berlin, Germany; ^3^Department of Economics, Freie Universitaet Berlin, Berlin, Germany; ^4^Faculty of Economics, Keio University, Tokyo, Japan

**Keywords:** COVID-19, preventive health behavior, vaccination, social norms, Japan, Germany, mandatory vaccination, SARS-CoV-2

## Abstract

**Background:**

According to a recent paper by Gelfand et al., COVID-19 infection and case mortality rates are closely connected to the strength of social norms: “Tighter” cultures that abide by strict social norms are more successful in combating the pandemic than “looser” cultures that are more permissive. However, countries with similar levels of cultural tightness exhibit big differences in mortality rates. We are investigating potential explanations for this fact. Using data from Germany and Japan—two “tight” countries with very different infection and mortality rates—we examined how differences in socio-demographic and other determinants explain differences in individual preventive attitudes and behaviors.

**Methods:**

We compared preventive attitudes and behaviors in 2020 based on real-time representative survey data and used logit regression models to study how individual attitudes and behaviors are shaped by four sets of covariates: individual socio-demographics, health, personality, and regional-level controls. Employing Blinder-Oaxaca regression techniques, we quantified the extent to which differences in averages of the covariates between Japan and Germany explain the differences in the observed preventive attitudes and behaviors.

**Results:**

In Germany and Japan, similar proportions of the population supported mandatory vaccination, avoided travel, and avoided people with symptoms of a cold. In Germany, however, a significantly higher proportion washed their hands frequently and avoided crowds, physical contact, public transport, peak-hour shopping, and contact with the elderly. In Japan, a significantly higher proportion were willing to be vaccinated. We also show that attitudes and behaviors varied significantly more with covariates in Germany than in Japan. Differences in averages of the covariates contribute little to explaining the observed differences in preventive attitudes and behaviors between the two countries.

**Conclusion:**

Consistent with tightness-looseness theory, the populations of Japan and Germany responded similarly to the pandemic. The observed differences in infection and fatality rates therefore cannot be explained by differences in behavior. The major difference in attitudes is the willingness to be vaccinated, which was much higher in Japan. Furthermore, the Japanese population behaved more uniformly across social groups than the German population. This difference in the degree of homogeneity has important implications for the effectiveness of policy measures during the pandemic.

## Introduction

**Figure 1:**
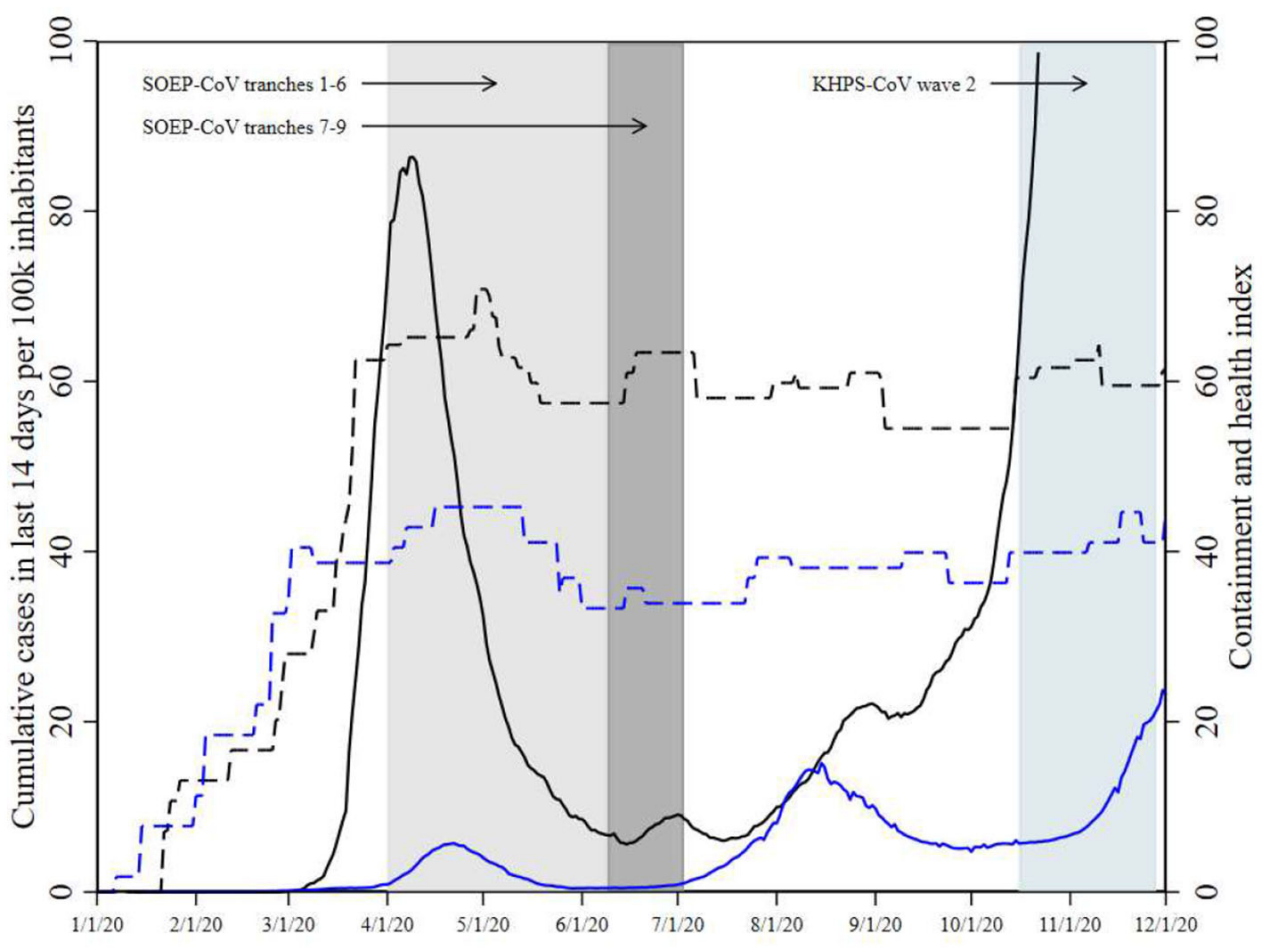
COVID-19 infections and government response during the survey periods. Data are from Nippon Hoso Kyokai, Robert-Koch Institut, and (2). Japanese data are in blue, German data in black. Solid lines indicate COVID-19 infections, dashed lines indicate the containment and health index. German infection data are only shown up to a value of 100 to ease comparison to the Japanese data. Fieldwork for SOEP-CoV was conducted in several tranches staggered over time, each representing a random sample. Tranches 7-9 of SOEP-CoV are shown separately (see Section 2), as attitudes toward vaccination were only surveyed only in these tranches.

Infection and case fatality rates for COVID-19 differ widely between countries. One variable that might be proposed to explain this cross-country variation is GDP. Although many health indicators do correlate with GDP [see, e.g., (1)], the case is less clear when it comes to the incidence of COVID-19. Consider Japan and Germany, for instance: Japan has a per capita GDP of about 40,000 US dollars, and as of the end of August 2021, it recorded around 1.6 million COVID-19 infections (1.27% of the population) and about 16,000 deaths (0.01% of the population). Germany has a similar per capita GDP of about 46,000 US dollars but recorded around 4 million infections (5% of the population) and 93,000 deaths (0.11% of the population). These differences are significant, and all the more surprising as Japan has a significantly older and more vulnerable population than Germany. In addition, the Japanese government imposed fewer and less restrictive legal measures to control the spread of the disease than the German government (see Figure 1).

What explains the observed heterogeneity in COVID-19 infections and deaths between countries? Many explanations have been put forward and are still being examined (3–9). We focus on the idea proposed by Gelfand et al. (10) that the strength of cultural norms—or, as they term it, cultural “tightness” or “looseness”—is associated with the success countries have in limiting infections and deaths from COVID-19. Tight cultures “have many strong norms and a low tolerance of deviant behavior,” whereas loose cultures “have weak social norms and a high tolerance of deviant behavior” (9, p. 1011). They regressed COVID-19 infection and mortality rates for about 50 countries on proxies for cultural tightness and looseness as well as a broad set of control variables and found that COVID-19 infection and mortality rates were about 5 to 9 times higher for loose countries than for tight countries.

According to (10), the crucial factor explaining infection and mortality rates is cultural. This is intuitively plausible: If people adhered closely to government rules on physical distancing and masking, there should indeed be fewer infections. Yet infection and mortality rates still vary widely between countries with similar level of cultural tightness—for instance, between Japan and Germany, both paradigmatically tight countries [(10), p. e137; (11), p. 1103; (12), p. 25].

To better understand why the incidence of infection differed so widely between Japan and Germany at the time of the study (2020), we examined the behavior of these two populations in more detail. We aimed to answer three research questions:

1. Did people in Japan and Germany reduce their social contacts, limit travel, and raise their hygiene standards to roughly the same degree?
2. Did people in Japan and Germany share the same attitudes toward vaccination, presumably the most effective tool for combating the pandemic?
3. To what extent can any differences in preventive behaviors and attitudes between the two countries be explained by differences in the behaviors of specific groups (e.g., age cohorts) and/or by differences in the composition of the two countries' populations?

Following tightness-looseness theory, we would expect the populations of Germany and Japan to behave similarly and have the same attitudes, other things being equal. If there are any differences in behavior or attitudes, these could be either due to unidentified cultural differences between Japanese and Germans, which would conflict with tightness-looseness theory, or be due to differences in the population structures. We investigated these three questions with data from two established, representative, large-scale panel studies: the Japan Household Panel Survey and the German Socio-Economic Panel, as well as their supplementary COVID-19 surveys (KPHS-CoV and SOEP-CoV). Figure 1 shows the field phases of the two surveys together with the country-specific COVID-19 daily infection rates and containment and health indices. SOEP-CoV was first fielded toward the end of the first wave of COVID-19 infections, and a second time during a low-incidence period in summer 2020. Wave 2 of KHPS-CoV was fielded during a period of low but increasing infection rates.

The policy recommendations in the two countries differed in 2020. In Japan, there was no formal lockdown (13, 14), and during the second wave (August 2020), the government did not declare a state of emergency but focused on economic countermeasures. People were asked to adopt behaviors and follow guidelines to prevent infections, but no legal sanctions or penalties were imposed for non-compliance. In Germany, a lockdown was imposed in late March 2020 (and lifted in early May) that severely limited individual mobility and business activities (2). People were no longer allowed to gather publicly in groups, meaning that schools, restaurants, and churches were closed. Larger private gatherings were also banned, and commercial facilities where physical proximity is inevitable, such as hairdressers and health clubs, had to close. Masks were required in grocery stores, public transport, and other indoor public spaces. A second lockdown was gradually introduced in November 2020. The first lockdown in Germany partially overlapped with the period of German data collection. Consistent with the described differences in policies, the Containment and Health Index (15) displayed in Figure 1 illustrates that the German response was stricter than the Japanese over the entire period of study.^1^

In brief, our results are as follows. In both Germany and Japan, the same percentage of the population favored mandatory vaccination, avoided travel, and avoided contact with people who were sick. However, in Germany, a higher percentage responded to the pandemic through preventive behaviors (e.g., handwashing), whereas in Japan, a higher percentage responded by being vaccinated. Furthermore, attitudes and behaviors differed substantially more by individual characteristics in Germany than in Japan. The large observed differences in infection and fatality rates cannot be explained by differences in behavior.

The paper is structured as follows. Section Methods describes our data sources and methods used. Section Results describes the results, and Section Discussion discusses these in more detail. In Section Conclusion, we conclude that behavioral differences are not the main driver of the differences in infection and mortality rates.

## Methods

The Japan Household Panel Survey (JHPS/KHPS) (17) is representative for Japan and covers about 5,470 adults (heads of households). The German Socio-Economic Panel (SOEP) is representative for Germany and is one of the longest-running panel surveys worldwide (18, 19). In 2020, the SOEP surveyed around 30,000 adults. Both studies contain a comparable set of variables on respondents' socio-demographic characteristics, health, personality traits, and attitudes. In 2020, both studies fielded supplementary surveys to gather information on the situations and experiences of respondents during the COVID-19 pandemic (18–20). The supplementary questions used in the two surveys matched each other well. The JHPS/KHPS conducted two waves of its supplementary survey on COVID-19 (KHPS-CoV): Wave 1 (3,891 respondents) was fielded in May and June 2020; Wave 2 (3,244 respondents) in October and November 2020 (in this paper, we focus on wave 2, as wave 1 did not survey attitudes towards vaccination). SOEP's COVID-19 study (SOEP-CoV) was conducted from April to July 2020 with about 6,700 respondents in nine time-staggered tranches.

### Information About Preventive Attitudes and Behaviors

KHPS-CoV and SOEP-CoV contained two modules central to the present paper. The first module asked for information on respondents' attitudes about vaccination against COVID-19. Since vaccines were not available in either country at the time of the survey, this module did not collect information on vaccination behavior but on attitudes. Respondents were asked (yes/no):

1. Whether they were willing to be vaccinated if they were offered a safe and effective vaccine
2. Whether they supported or opposed a policy of mandatory vaccination.

The second module asked respondents about their willingness to adhere (yes/no) to different preventive measures to avoid the spread of SARS-CoV-2.^2^ Respondents were asked whether they:

1. Avoided contact with elderly and chronically ill people
2. Avoided using public transportation
3. Refrained from travel, including domestic travel
4. Avoided peak-hour shopping
5. Avoided closed spaces, crowded places, and close-contact settings (“three Cs”)
6. Kept their distance from people with a cough, cold, or fever
7. Avoided physical contact, such as shaking hands
8. Washed their hands regularly.

In our analyses, responses to all items from the two modules were coded as indicator variables, with 1 indicating either that the respondent acted in the described manner or that the respondent was willing to be vaccinated or supported mandatory vaccination. Supplementary Table 1 in the Supplementary Material provides information on the content and coding of the related variables.

### Covariates

Our analysis included four sets of controls that could plausibly relate to cross-country variation in preventive attitudes and behaviors:

1. Sociodemographic characteristics: age, gender, an indicator for tertiary education, monthly household net income in 1,000 US dollars PPP-adjusted, presence of children in the household.
2. Health: self-assessed health status and the number of medical diseases associated with severe COVID-19 infections.
3. Personality: Big Five “OCEAN” personality traits (22): openness to experience, conscientiousness, extraversion, agreeableness, neuroticism.
4. Regional indicators: country indicator, region of residence (Germany: NUTS2, Japan: prefecture^3^), number of confirmed COVID-19 cases in the last 14 days per 100,000 inhabitants in the respective region, weekly change in this COVID-19 incidence, nominal GDP per capita in 1,000 US dollars PPP-adjusted, population density in people per square kilometer, unemployment rate.

All covariates except COVID-19 incidences refer to the period before the pandemic. Hence, they can be considered exogenous to the pandemic event. Supplementary Tables 2, 3 in the Supplementary Material provide content and coding of all covariates, which were harmonized before the analysis. The covariates do not include specific public appeals, recommendations, or even laws in the two countries. This is because none of the preventive behaviors we investigated were legally obligatory at the time of the interviews, but all were consistently recommended by the health authorities.

### Statistical Framework

Preventive attitudes and behaviors, our outcome variables, were reported only once by each respondent, so we cannot control for individual fixed effects. Univariate results for the outcome variables are presented as weighted means or percentages. The person weights account for imbalances between the sample and the respective population (23). To test for differences between the two countries, we ran Wald-tests (including a Bonferroni-Holm correction to counteract the problem of multiple comparisons). To statistically explain the relationship between the 10 outcome variables and the four sets of covariates, we estimated weighted logit regressions pooling the datasets of both countries. The purpose of the logit regressions was to estimate the probability that a respondent, *i*, with a certain set of characteristics, **X**_*i*_, will fall into one of two categories: the respondent's behavior or attitude is preventive (outcome variable equals 1) or is not preventive (outcome variable equals 0). To quantify differences between the two countries, the set of explanatory variables included, in addition to the set of characteristics, an interaction of each of those characteristics with a country dummy for Japan, **X**_*i*_×*D*^*JP*^, as well as the Japan dummy, *D*^*JP*^. If the regression coefficient pertaining to the interaction of a particular control variable with the Japan dummy was zero, then this control variable had the same influence on the outcome variable of interest in both countries. The coefficient pertaining to the Japan dummy described differences in behavior or attitudes between the two countries that could not be explained by observable characteristics.

In addition to different correlation patterns within each country, there may be a further cause for the differences between Japan and Germany: different endowments of the covariates such as age structures between the two countries (24). Hence, as an additional statistical device, we implemented a Blinder-Oaxaca decomposition (25).^4^ It decomposes the difference of the likelihoods between the two countries into two parts: the part that is due to differences in the mean values of the explanatory variables and the part that is due to differences in the correlations of the dependent variables with the explanatory variable. The first part is commonly termed “explained difference,” the second part the “unexplained difference,” as it also captures unobserved effects. Subtracting the differences in outcome that arise due to different mean covariates (i.e., the *explained* difference) from the *observed* difference in outcomes gives a *hypothetical* difference in outcomes: the difference in outcomes that would obtain if, ceteris paribus, the two countries had the same mean covariates.

## Results

### Preventive Attitudes and Behaviors

Table 1 compares preventive attitudes and behaviors in Germany and Japan. Columns 2 and 3 give the weighted share of individuals responding “yes” to the respective question. Column 4 lists the F-statistics resulting from a Wald-test for difference in means. Column 5 gives Bonferroni-Holm-adjusted *p*-values. Columns 6 and 7 provide the number of observations for each item.^5^ In SOEP-CoV, some topical modules were randomly assigned to respondents, explaining why responses to the two vaccination questions are lower.

**Table 1:** Preventive attitudes and behaviors by country.

**Variables**	**Sample average (%)**		**Wald-test for difference**		**Sample size**	
	**Germany**	**Japan**	***F***	***p* (Holm)**	**Germany**	**Japan**
Preventive attitudes						
Willingness to get vaccinated	69.840	85.430	44.709	0.000	812	2,491
Supporting mandatory vaccination policy	48.650	44.430	2.127	0.435	822	3,080
Preventive activities						
Avoiding the elderly	78.890	64.390	125.194	0.000	6,694	3,058
Avoiding public transport	83.320	70.980	101.378	0.000	6,694	3,065
Avoiding travelling	89.600	88.780	0.781	0.377	6,694	3,063
Avoiding shopping at peak hours	81.610	73.930	40.423	0.000	6,694	3,067
Avoiding crowds	94.910	91.530	20.955	0.000	6,694	3,073
Avoiding persons with symptoms of a cold	91.350	92.080	0.818	0.731	6,694	3.060
Avoiding physical contact	94.480	92.350	7.930	0.019	6,694	3,070
Washing hands	96.820	86.590	140.751	0.000	6,692	3,070

*Data are from SOEP-CoV and KHPS-CoV. All numbers in % and weighted. F stands for F-statistic, p (Holm) stands for p-value after applying the Bonferroni-Holm adjustment*.

The vast majority of respondents in Japan and Germany said they engaged in preventive behavior and avoided risky activities. In both samples, more than 60% reported that they avoided contact with elderly and chronically ill people; more than 70% reported that they avoided public transportation, travel, and peak-hour shopping; 90% or more avoided crowds, kept distance from people with symptoms of a cold, avoided physical contact, and washed their hands regularly.

Overall, the proportions of respondents who engaged in preventive behaviors across the eight dimensions described were very similar in Japan and Germany, although overall slightly higher in Germany. Nevertheless, some significant differences became evident: Respondents in the Japanese sample were much more willing to be vaccinated (about 85%) than those in the German sample (70%). Support for mandatory vaccination was just under 50% in both countries and did not differ statistically between them.

### Characteristics of the Two Populations

To better explain what is driving these overall results, Table 2 shows summary statistics for the four sets of covariates following the structure of Table 1. We found significant differences between the two countries for 14 out of 17 covariates. In terms of socio-demographic characteristics, the Japanese sample population had a similar proportion of women and was of similar average age to the German sample. The Japanese sample was more educated, had lower household income, and contained a smaller share of households with children below the age of 17. Respondents in both countries assessed their own health as quite good, and the difference was not significant, although the number of medical diseases associated with complicated COVID-19 infections was significantly higher in Germany than in Japan. In terms of personality, respondents in Germany were more extroverted, more conscientious, more open, less neurotic, and more agreeable than those in Japan. Respondents in Germany lived in regions that had a higher level of infections but where infections were largely on a downward trend (see also Figure 1). German respondents also lived in regions with higher GDP per capita, lower population density, and higher unemployment rates before the pandemic than the Japanese respondents.

**Table 2:** Covariates by country.

**Variables**	**Sample average**		**Wald-test for difference**		**Sample size**	
	**Germany**	**Japan**	***F***	***p (Holm)***	**Germany**	**Japan**
Sociodemographic						
Female (%)	50.840	51.570	0.257	1.000	6,688	3,080
Age	53.080	53.450	0.436	1.000	6,683	3,080
Tertiary education (%)	25.560	29.340	8.777	0.018	6,508	3,080
Net monthly household income (k USD PPP)	4.040	3.900	3.975	0.185	6,535	2,665
Share with children of age <17 (%)	24.100	21.020	6.308	0.060	6,499	3,080
Health						
Self-assessed health (1 to 5)	2.630	2.620	0.142	0.707	6,643	3,080
Number of risky diseases	0.530	0.380	51.717	0.000	6,200	3,080
Big Five (1 to 7)						
Extraversion	4.920	3.860	859.254	0.000	6,406	3,080
Conscientiousness	5.770	4.070	3,133.721	0.000	6,403	3,080
Openness to experience	4.860	4.140	482.167	0.000	6,384	3,080
Neuroticism	3.530	4.150	330.077	0.000	6,419	3,080
Agreeableness	5.400	2.950	7,698.050	0.000	6,413	3,073
Regional (D: NUTS2, J: prefecture)						
COVID-19 cases in 14 days before interview (per 100k inhabitants in region)	33.370	6.280	1,882.806	0.000	6,694	3,011
Weekly change of COVID-19 cases on day of interview (%)	−16.060	33.720	76.989	0.000	6,694	2,939
Nominal GDP per capita (k USD PPP)	54.960	42.960	1,084.644	0.000	6,694	3,063
Population density (k people/sqkm)	0.560	1.900	721.120	0.000	6,694	3,063
Unemployment rate (%)	5.040	2.260	6,666.856	0.000	6,694	3,063

*Data are on the individual level from SOEP-CoV and KHPS-CoV. For regional data sources, please see Supplementary Table 4 in the Supplementary Material. Regional data for Germany refer to NUTS2 regions, for Japan to prefectures. All numbers are weighted. All variables apart from COVID-19 cases refer to pre-2020 levels. Section 1 of the Supplementary Material provides definitions of all the variables and details on the construction of the Big Five. F stands for F-statistic, p (Holm) stands for p-value after applying the Bonferroni-Holm adjustment*.

### Preventive Attitudes and Behaviors—The Role of Covariates

Tables 3.1, 3.2 quantify the role of respondents' characteristics described in the preceding section for the respective preventive attitudes and behaviors.^6^ For each covariate, two rows represent the regression coefficient for the German sample (D) and how the coefficient *differs* for the Japanese sample (indicated by Δ J).

**Table 3.1:** Regression coefficients of covariates on attitudes and behavior (1).

**Covariates**	**Voluntary vacc**	**Mandatory vacc**	**Avoid elderly**	**Avoid transport**	**Avoid travelling**
Japan	4.268^***^	0.104	0.229	−0.848	−0.540
Sociodemographic					
Female (D)	−0.606^**^	−0.197	0.069	0.097	0.303^*^
Δ Female (J)	0.465	−0.131	0.064	0.109	0.239
Age (D)	0.030^***^	0.021^**^	−0.008^**^	−0.005	−0.001
Δ Age (J)	−0.040^***^	−0.018^*^	0.005	0.015^**^	0.018^**^
Tertiary education (D)	1.464^***^	0.112	−0.022	−0.309^**^	−0.485^***^
Δ Tertiary education (J)	−1.336^***^	−0.297	0.264	−0.045	0.050
Net monthly household income (D)	0.158^**^	−0.016	0.051^*^	0.111^**^	0.020
Δ Net monthly household income (J)	−0.152^**^	0.030	−0.067^**^	−0.144^***^	−0.064
Share with children of age <17 (D)	0.386	0.348	−0.151	−0.013	0.105
Δ Share with children of age <17 (J)	−0.612	−0.359	0.274	0.505^**^	−0.016
Health					
Self-assessed health (D)	0.102	−0.006	−0.030	−0.120^*^	−0.112
Δ Self-assessed health (J)	0.024	0.003	−0.013	0.150^*^	0.218^*^
Number of risky diseases (D)	−0.001	0.228	0.065	0.120	0.045
Δ Number of risky diseases (J)	0.111	−0.127	−0.103	−0.120	−0.105
Big Five					
Extraversion (D)	−0.387^***^	−0.091	−0.036	−0.008	0.079
Δ Extraversion (J)	0.361^**^	0.071	0.083	−0.075	0.057
Conscientiousness (D)	−0.295^*^	0.015	0.082	0.193^***^	0.012
Δ Conscientiousness (J)	0.315^*^	−0.051	−0.201^***^	−0.203^**^	−0.013
Openness to experience (D)	0.334^**^	0.066	0.047	0.001	0.055
Δ Openness to experience (J)	−0.328^*^	−0.098	−0.109	0.036	0.023
Neuroticism (D)	−0.139	−0.325^**^	−0.046	0.170^**^	0.179^*^
Δ Neuroticism (J)	−0.091	0.194	−0.048	−0.263^***^	−0.235^*^
Agreeableness (D)	0.175	0.001	0.079	0.055	0.019
Δ Agreeableness (J)	−0.209	−0.010	−0.023	−0.128	−0.138
Regional (D: NUTS2, J: prefecture)					
COVID-19 cases in last 14 days (D)	0.042^**^	−0.010	0.009^***^	0.008^***^	0.015^***^
Δ COVID-19 cases in last 14 days (J)	−0.079^***^	0.028	0.009	−0.020	0.008
Weekly change COVID-19 cases (D)	−0.220	0.285	−0.276^**^	−0.031	−0.474^***^
Δ Weekly change COVID-19 cases (J)	0.244	−0.273	0.251^*^	0.041	0.479^***^
Nominal GDP per capita (D)	0.004	−0.014	0.004	−0.009	−0.005
Δ Nominal GDP per capita (J)	0.016	0.021	−0.008	0.021^**^	0.017
Population density (D)	−0.412^*^	−0.053	−0.132^*^	−0.154^**^	0.028
Δ Population density (J)	0.394^*^	−0.027	0.188^**^	−0.044	−0.147
Unemployment rate (D)	0.151	0.052	0.071^*^	0.038	0.084
Δ Unemployment rate (J)	−0.112	0.032	−0.197	−0.172	−0.093
**Sample size**	**2,750**	**3,237**	**8,357**	**8,367**	**8,364**

*Data are on the individual level from SOEP-CoV and KHPS-CoV. For regional data sources, please see Supplementary Table 4 of the Supplementary Material. All regression coefficients from weighted regressions. Coefficient for the intercept is not shown. The Big Five and self-assessed health are measured in standard deviations. Section 1 of the Supplementary Material provides definitions of all the variables and details on the construction of the Big Five. Rows with a (D) give the coefficients for the German sample. Rows with a Δ show how the coefficients for the Japanese sample differ*.

* *p <0.1*,

** *p <0.05*,

*** *p <0.01. The green (red) colors indicate positive (negative) point estimates, which are different from zero at a significance level of 10, 5 or 1 percent*.

**Table 3.2:** Regression coefficients of covariates on attitudes and behavior (2).

**Covariates**	**Avoid peak shopping**	**Avoid crowds**	**Avoid symptoms**	**Avoid contact**	**Wash hands**
Japan	0.595	−1.160	0.002	0.162	−0.697
Sociodemographic					
Female (D)	0.389^***^	0.376^*^	0.107	−0.015	0.459
Δ Female (J)	−0.122	0.380	0.524^**^	0.685^**^	0.272
Age (D)	0.009^**^	0.001	−0.022^***^	0.014^**^	0.002
Δ Age (J)	−0.013^**^	0.006	0.009	0.001	−0.014
Tertiary education (D)	−0.114	−0.149	0.180	0.090	0.243
Δ Tertiary education (J)	0.153	0.599^*^	−0.171	0.257	−0.422
Net monthly household income (D)	0.032	−0.005	0.029	0.152^**^	0.080
Δ Net monthly household income (J)	−0.072^**^	−0.025	−0.051	−0.113	−0.094
Share with children of age <17 (D)	−0.096	−0.052	−0.516^**^	−0.084	−0.066
Δ Share with children of age <17 (J)	−0.102	0.064	0.607^*^	0.464	0.279
Health					
Self-assessed health (D)	−0.170^***^	−0.061	−0.020	−0.058	0.017
Δ Self-assessed health (J)	0.243^***^	−0.036	0.031	0.248	−0.146
Number of risky diseases (D)	0.226^***^	0.211	0.279^**^	0.072	−0.051
Δ Number of risky diseases (J)	−0.253^**^	−0.183	−0.015	0.058	0.267
Big Five					
Extraversion (D)	−0.077	−0.012	0.018	−0.302^***^	0.383^***^
Δ Extraversion (J)	0.136	0.108	−0.051	0.384^**^	−0.264
Conscientiousness (D)	0.087	0.064	0.164^**^	0.233^**^	0.080
Δ Conscientiousness (J)	−0.147^*^	−0.224	−0.305^**^	−0.334^**^	−0.322^*^
Openness to experience (D)	0.201^***^	0.094	−0.061	0.013	0.113
Δ Openness to experience (J)	−0.271^***^	−0.045	0.088	−0.034	−0.140
Neuroticism (D)	0.164^**^	0.322^**^	0.169^**^	0.291^***^	0.096
Δ Neuroticism (J)	−0.195^**^	−0.338^**^	−0.248^*^	−0.429^***^	−0.133
Agreeableness (D)	0.017	0.068	0.215^***^	0.110	0.358^**^
Δ Agreeableness (J)	−0.044	−0.048	−0.270^**^	−0.160	−0.357^**^
Regional (D: NUTS2, J: prefecture)					
COVID-19 cases in last 14 days (D)	0.008^***^	0.012^***^	0.004^*^	0.013^***^	0.002
Δ COVID-19 cases in last 14 days (J)	0.012	−0.026	0.029	0.028	0.073^**^
Weekly change COVID-19 cases (D)	−0.004	−0.034	0.251	−0.348^*^	0.168
Δ Weekly change COVID-19 cases (J)	0.021	0.060	−0.284	0.325^*^	−0.211
Nominal GDP per capita (D)	0.002	−0.004	0.002	0.003	−0.006
Δ Nominal GDP per capita (J)	0.004	0.018	0.008	−0.005	0.001
Population density (D)	−0.185^**^	−0.184	−0.220^**^	−0.204	−0.318^*^
Δ Population density (J)	0.168^*^	0.205	0.183	0.163	0.243
Unemployment rate (D)	0.040	0.070	0.123^*^	0.123^*^	0.163
Δ Unemployment rate (J)	0.023	−0.113	−0.283	0.051	−0.126
**Sample size**	**8,365**	**8,370**	**8,361**	**8,368**	**8,367**

*Data are on the individual level from SOEP-CoV and KHPS-CoV. For regional data sources, please refer to Supplementary Table 4 in the Supplementary Material. All regression coefficients from weighted regressions. Coefficient for the intercept is not shown. The Big Five and self-assessed health are measured in standard deviations. Section 1 of the Supplementary Material provides definitions of all the variables and details on the construction of the Big Five. Rows with a (D) give the coefficients for the German sample. Rows with a Δ show how the coefficients for the Japanese sample differ*.

* *p <0.1*,

** *p <0.05*,

*** *p <0.01. The green (red) colors indicate positive (negative) point estimates, which are different from zero at a significance level of 10, 5 or 1 percent*.

Regarding the attitude toward voluntary vaccination, several individual-level characteristics are important predictors in Germany (age, education, income, and some character traits) but not in Japan. For attitudes towards mandatory vaccination schemes, there is almost no variation across individual characteristics in either country. Only age made people slightly more inclined to endorse this policy in Germany.

Regarding preventive behaviors, older people in Germany, in general, were more likely to engage in preventive behavior, but this association was not very pronounced in Japan. In Japan, it was women who were more likely to engage in risk-reducing behavior. With respect to health variables, people at higher risk of severe COVID-19 outcomes in both countries were slightly more likely to report risk-reducing behavior. With respect to personality traits, more conscientious and more neurotic people were more likely to report risk-reducing behavior—at least in Germany—whereas more neurotic people in both countries were less supportive of mandatory COVID-19 vaccination. With respect to regional characteristics, in both countries, a higher level of COVID-19 infections in the 14 days before the interview was associated with more preventive behavior. In Germany, this number was also positively correlated with the willingness to be vaccinated. We also see that, in Germany, compared to Japan, people in regions with higher unemployment rates and lower population density were more likely to adopt risk-reducing attitudes and behaviors.

Overall, many covariates do not have a systematic influence on preventive attitudes and behaviors. Most individual-level differences observed in Germany are also not present in Japan. Finally, only for the willingness to be vaccinated did we observe a significant difference between the German and the Japanese sample: Japanese respondents were more likely to be vaccinated voluntarily than German respondents, controlling for all the covariates.

### Preventive Attitudes and Behaviors—The Role of Country-Specific Mean Levels of Covariates

Tables 4.1, 4.2 give the results of a Blinder-Oaxaca decomposition (25). Each column gives the raw difference in weighted averages of the two countries and the level of the difference if Germany had exactly the same mean values of the covariates (endowments) as Japan. The objective is to determine whether the overall differences in preventive attitudes and behaviors can be explained by differences in the endowments of covariates. If so, it could be these differences rather than deeper differences in the respective cultures not captured by tightness-looseness theory that might explain the differences in infection and fatality rates.

**Table 4.1:** Contribution of differential endowments to differential attitudes and behaviors (1).

	**Voluntary vacc**.	**Mandatory vacc**.	**Avoid elderly**	**Avoid transport**	**Avoid travelling**
Germany	68.7	46.5	78.5	83.0	89.8
Japan	86.1	45.6	64.1	70.9	88.6
Raw difference (D-J)	−17.4^***^	0.9	14.4^***^	12.1^***^	1.1
Difference after adjusting covariates	−39.3	−0.5	−0.7	4.5	−6.7
Explained difference: Sociodemographic					
Female	−0.3	−0.5	−0.0	−0.0	−0.0
Age	0.1	0.6	0.1	0.0	0.0
Tertiary education	1.5^*^	0.3	−0.0	−0.2^*^	−0.2^**^
Net monthly household income	−0.7	0.2	−0.1	−0.2	−0.0
Share with children of age <17	0.0	−0.1	0.0	0.0	−0.0
Explained difference: Health					
Self-assessed health	−0.1	0.0	0.0	0.1	0.0
Number of risky diseases	0.0	−2.4	−0.2	−0.3	−0.1
Explained difference: Big Five					
Extraversion	−0.5	−0.5	−0.0	−0.0	0.0
Conscientiousness	−1.2^*^	0.2	0.2	0.4^***^	0.0
Openness to experience	0.2	0.2	0.0	0.0	0.0
Neuroticism	−0.1	−0.3	−0.0	0.1	0.1
Agreeableness	0.2	0.0	0.1	0.1	0.0
Explained difference: Regional					
COVID-19 cases in last 14 days	−1.1^*^	0.9	−4.6^***^	−3.4^***^	−4.0^***^
Weekly change COVID-19 cases	−0.8	3.7	−2.4^**^	−0.2	−2.3^**^
Nominal GDP per capita	−0.9	10.2	−0.7	1.7^*^	0.6
Population density	−10.8^*^	−4.8	−3.7^*^	−3.8^*^	0.4
Unemployment rate	−7.5	−9.0	−3.7	−1.8	−2.4
**Sample size**	**2,750**	**3,237**	**8,357**	**8,367**	**8,364**

*Data are on the individual level from SOEP-CoV and KHPS-CoV. For regional data sources, please refer to Supplementary Table 4 in the Supplementary Material. Numbers are reported in percentage points and are weighted. “Raw difference” differs from the difference reported in Table 1, as we only consider the restricted sample, for which all covariates are non-missing here. We do not provide significance levels in “Difference after adjusting covariates” as this is not estimated but computed algebraically. The Big Five and self-assessed health are measured in standard deviations. Section 1 of the Supplementary Material provides definitions of all the variables and details on the construction of the Big Five*.

* *p <0.1*,

** *p <0.05*,

*** *p <0.01. The green (red) colors indicate positive (negative) point estimates, which are different from zero at a significance level of 10, 5 or 1 percent*.

**Table 4.2:** Contribution of differential endowments to differential attitudes and behaviors (2).

	**Avoid peak shopping**	**Avoid crowds**	**Avoid symptoms**	**Avoid contact**	**Wash hands**
Germany	82.0	95.2	91.7	94.4	97.2
Japan	74.2	91.7	92.5	93.0	87.0
Raw difference (D-J)	7.7^***^	3.5^***^	−0.8	1.4^*^	10.2^***^
Difference after adjusting covariates	−3.4	−1.3	−8.6	−7.9	6.2
Explained difference: Sociodemographic					
Female	−0.0	−0.0	−0.0	0.0	−0.0
Age	−0.1	−0.0	0.2	−0.1	−0.0
Tertiary education	−0.1	−0.0	0.1	0.0	0.1
Net monthly household income	−0.1	0.0	−0.0	−0.2	−0.1
Share with children of age <17	0.0	0.0	0.0	0.0	0.0
Explained difference: Health					
Self-assessed health	0.1	0.0	0.0	0.0	−0.0
Number of risky diseases	−0.6^**^	−0.2	−0.5^**^	−0.1	0.0
Explained difference: Big Five					
Extraversion	−0.0	−0.0	0.0	−0.0	0.0
Conscientiousness	0.2	0.1	0.3^*^	0.3^**^	0.1
Openness to experience	0.0	0.0	−0.0	0.0	0.0
Neuroticism	0.1	0.1	0.0	0.1	0.0
Agreeableness	0.0	0.0	0.2	0.1	0.1
Explained difference: Regional					
COVID-19 cases in last 14 days	−3.6^***^	−2.0^*^	−1.4	−2.7^**^	−0.2
Weekly change COVID-19 cases	−0.0	−0.1	1.4	−1.3^*^	0.4
Nominal GDP per capita	−0.3	0.3	−0.2	−0.2	0.4
Population density	−4.7^**^	−1.7	−3.8	−2.4	−2.4
Unemployment rate	−1.9	−1.2	−4.0	−2.7	−2.3
**Sample size**	**8,365**	**8,370**	**8,361**	**8,368**	**8,367**

*Data on individual level from SOEP-CoV and KHPS-CoV. For regional data sources, please refer to Supplementary Table 4 of the Supplementary Material. Numbers are reported in percentage points and are weighted. “Raw difference” differs from the difference reported in Table 1, as we only consider the restricted sample, for which all covariates are non-missing here. We do not provide significance levels in “Difference after adjusting covariates” as this is not estimated but computed algebraically. The Big Five and self-assessed health are measured in standard deviations. Section 1 of the Supplementary Material provides definitions of all the variables and details on the construction of the Big Five*.

* *p <0.1*,

** *p <0.05*,

*** *p <0.01. The green (red) colors indicate positive (negative) point estimates, which are different from zero at a significance level of 10, 5 or 1 percent*.

To give an example, consider the outcome “avoiding contact with the elderly.” The share of individuals who reported avoiding contact was 14.4 percentage points higher in Germany than in Japan (raw difference; Table 4.1, column 3, line 3). If Germany had the same mean level of covariates as Japan based on correlations of all the covariates with avoiding the elderly (as displayed in Table 3.1)—that is, if the two countries had the same proportion of women, the same mean level of education, etc.—the raw difference in avoiding contact with the elderly would change to −0.7 percentage points and thus almost disappear. The difference of 15.1 (14.4+0.7) between these two numbers (not shown in the table) was therefore due to the difference in the average covariates and their impact on the outcome as represented by our model. Japanese and Germans would behave almost identically if all the covariates were identical.

Overall, for four out of the seven preventive attitudes and behaviors for which there was a significant difference between the two countries, differential levels of endowments have a significant overall effect on the difference: willingness to be vaccinated, avoiding contact with the elderly, avoiding peak-hour shopping, and avoiding contact in closed spaces. In the case of willingness to be vaccinated, alignment of means in the covariates would further increase the observed difference, but for the other three variables, the difference in mean covariates helped to close the observed gap in outcomes. In other words, if circumstances in Germany were similar to those in Japan, the gap in willingness to be vaccinated would increase further. For the other variables, the outcomes would converge.

The regional characteristics are the best predictor for differential preventive attitudes and behaviors on the whole. If regional characteristics predict a significant difference in outcomes, they would point to the expectation that the German population is more preventive than the Japanese population. Across all covariates, the number of COVID-19 cases in the last 14 days had the most consistent impact on the overall results: It affected 7 out of 10 outcomes. Population density also matters.

Differences in sociodemographic, health, and personality covariates are a weaker predictor for differential behaviors and attitudes. For these categories of covariates, only one variable in each category significantly helped to explain observed differences. For the sociodemographic variables, this variable was tertiary education. For the health categories, it was the number of risky diseases. For the Big Five psychological characteristics, it was conscientiousness. The other variables did not significantly impact the difference between Japan and Germany.

## Discussion

The large similarities in preventive behavior in Germany and Japan—both with and without control variables—correspond to the similar positioning of both countries on the cultural tightness scale and confirm the starting hypothesis of (10). The differences in infection and fatality rates between the two countries cannot be explained by differences in behavior, however, which is in conflict with the main thesis of (10). Some evidence, indeed, points to Japanese culture being even “tighter” than German culture: The behavior of the Japanese population was consistently more homogeneous than that of the German population. Respondents in the Japanese sample reported very similar behavior, whether they were male or female, had a higher or lower level education, etc. This finding is consistent with Japan having stronger norms than Germany, suppressing individual differences in the willingness to be vaccinated by socio-economic status or individual personality traits. The fact that the Japanese population was so much more willing to be vaccinated also fits this picture, though recent research seems to indicate the willingness to get vaccinated does not seem to depend on the tightness score of a country (26). Be that as it may, these minor behavioral differences do not explain why the pandemic affected Japan and Germany so differently.

Comparing our figures to the earlier analysis in Muto et al. (27), it also seems that Japanese people have increased their preventive efforts since the first wave, although in recent months, their willingness to be vaccinated seems to have declined (28). Traditionally, vaccine uptake is lower in Japan than what our figures suggest (29), although it has been suggested that this might not apply to the case of COVID-19 (30, 31), which is borne out by the current rate of vaccination of almost 80% as of the end of January 2022.^7^ The German data were in line with COVID-19 Snapshot Monitoring (32) and even slightly higher at the end of January 2022 (about 73%).^8^

The most important predictors of preventive attitudes and behaviors in our analysis were regional variables, in particular population density and COVID-19 cases. Comparing our findings on these regional variables to the literature yields a range of interesting observations. First, our results on the role of population density conflict with other stylized facts of tightness-looseness theory. According to Gelfand, higher population density is typically associated with a tighter culture (11, pp. 59ff.). Other authors controlling for similar individual-level characteristics to those used here have reported an association between population density and preventive behaviors during the COVID-19 pandemic in the United States (33), China (34), and Vietnam (35). While we found that population density does not play a role in Japan, our results show that more densely populated areas of Germany are “looser” and less cautious when it comes to preventive behavior, though the theoretical prediction would have been the exact opposite. Second, cases of COVID-19 in the respondents' regions matter for their behavior. This is very much in line with the health belief model (36) and protection motivation theory (37, 38), as incidence rates may directly influence perceptions of the dangerousness of the virus.

## Conclusion

To find ways to combat the spread of COVID-19 or comparable viruses, we need to understand how people react to the guidelines and rules introduced by public health authorities. If people behave responsibly and comply with sensible recommendations, fewer restrictions will have to be put in place. How have national populations differed in their preventive behavior and attitudes toward the pandemic?

We investigated two countries which, despite being thousands of miles apart, share an important cultural feature: In both Japan and Germany, social norms are taken seriously, and non-compliers are punished informally—both cultures are very “tight” in the terminology of (11). Indeed, generally speaking, this cultural fact seems to go a long way in explaining why some countries deal better with the COVID-19 pandemic than others (10). We attempted to refine this observation further with more detailed individual data on socio-demographic characteristics, health, personality traits, and regional-level controls than previous research has done. What impact do these factors have, and are cultural factors really that important? The comparison between these two countries is of particular interest because the numbers of COVID-19 infections and deaths have differed significantly between them.

Our results indicate that individual behavior does not explain this difference, since, as expected, the populations of Japan and Germany did behave similarly in reaction to the pandemic. We controlled for differences in the two populations, both at the individual as well as at the regional level. Japanese and German people behaved almost identically when comparing individuals with the same socio-economic background, age, etc. The major difference that our model did not explain was the willingness to be vaccinated, which was much higher in Japan than in Germany. We also found that people in Japan behaved more uniformly than those in Germany, who, on average, complied with the recommendations slightly better.

Our analysis does not shed light on why Germany has been so much more affected by the pandemic than Japan. Factors not represented in our explanatory variables must be taken into account to explain these differences. These factors might well also be cultural, but they would not be covered by the tightness-looseness distinction (39). However, other factors seem to be more promising for future research, though it is hard to say which (3–9).

Our analysis has some methodological limitations due to the data sources available to us. Future research should consider changes in attitudes from one wave of the pandemic to the next, evolution of the virus strain and corresponding changes in its perceived dangerousness, as well as changes in attitudes and behavior due to seasonal factors (people would tend to be more cautious in winter than in summer). Our analysis also needs to assume that neither norms nor compliance changed over the period of investigation. There is, of course, also the possibility that the self-reported behavior we analyzed does not correspond 1:1 to respondents' actual behavior. Furthermore, our analysis did not consider other factors such as political orientation or media consumption (40), which could impact several of the attitudes we considered. Finally, it would be worthwhile to examine in more detail which social groups contribute to spreading the virus within their respective countries and whether differences in compliance could explain this, even if the aggregated national values do not differ between Japan and Germany.

## Data Availability Statement

Data are available from the German Socio-economic Panel Study (SOEP) due to third party restrictions (for requests, please contact soepmail@diw.de). The SOEP is made available free of charge to universities and research institutes for research and teaching purposes. The direct use of SOEP data is subject to the strict provisions of German data protection law. Therefore, signing a data distribution contract is a precondition for working with SOEP data. The data distribution contract can be requested with a form. The form is provided here: http://www.diw.de/documents/dokumentenarchiv/17/diw_01.c.88926.de/soep_application_contract.pdf. For further information the SOEPhotline at either soepmail@diw.de or +49 30 89789- 292 can be contacted. JHPS/KHPS data are available from Panel Data Research Center at Keio University under some restrictions (https://www.pdrc.keio.ac.jp/en/paneldata/datasets/jhpskhps/). The KHPS is made available free of charge to universities and research institutes for research and teaching purposes. The direct use of KHPS/JHPS data is subject to some conditions. Signing a data distribution contract and submitting research proposals are required for the access.

## Author Contributions

CS, CS-P, and TO have designed the questionnaires. CS, CS-P, TO, TR, and DG have analysed and interpreted the data, written the article, and approved the final version.

## Funding

The data collection of the SOEP-CoV Study was financially supported by the German Federal Ministry of Education and Research. We acknowledge support by the KIT-Publication Fund of the Karlsruhe Institute of Technology. The funders had no role in study design, data collection and analysis, decision to publish, or preparation of the manuscript.CS acknowledges funding from the German Research Foundation (SCHR 1498/7-1). The data collection of the KHPS Study was financially supported by Grant-in-Aid for Specially Promoted Research (Japan Society for the Promotion of Science, JSPS). We also acknowledge financial support by Grants-in-Aid for Scientific Research (Japan Society for the Promotion of Science, JSPS) (19H01487).

## Conflict of Interest

The authors declare that the research was conducted in the absence of any commercial or financial relationships that could be construed as a potential conflict of interest.

## Publisher's Note

All claims expressed in this article are solely those of the authors and do not necessarily represent those of their affiliated organizations, or those of the publisher, the editors and the reviewers. Any product that may be evaluated in this article, or claim that may be made by its manufacturer, is not guaranteed or endorsed by the publisher.

## Footnotes

^1^Face masks have been worn regularly in Japan, especially in winter, since the flu pandemic of 1918-1920 (16). The customary greeting in Japan is a bow as opposed to a handshake or hug. Japan is thus unique in having social customs that are inherently preventive of infections, which meant that no government regulations to enforce such behaviors were needed in the COVID-19 pandemic. As such, at least in certain important respects, the demands on the Japanese population were not as strict as the demands on the German population.

^2^In the slightly more precise terminology of (21), some of these behaviors are avoidant and some are preventive.

^3^NUTS (Nomenclature des unités territoriales statistiques) is a European geocode standard used to subdivide the territory of the EU and UK at different levels of granularity for statistical and analytical purposes. NUTS2 is the classification of medium granularity and divides Germany into 38 regions. For comparison, Japan has 47 prefectures.

^4^Section Description of Blinder-Oaxaca Decomposition Method of the Supplementary Material gives a more detailed and formal explanation of the Blinder-Oaxaca decomposition in the context of binary dependent variables.

^5^In SOEP-CoV, questions on preventive attitudes were only asked in tranches 7 to 9 (dark gray area of Figure 1). Therefore, the sample size is much smaller than for the preventive behaviors. Also note that 615 respondents answered “I don't know” in KHPS-CoV to the question about their willingness to be vaccinated, explaining the lower sample size in the first row of the last column.

^6^As a robustness check, we estimated four further specifications, each using a single set of covariates. The results support the general results of the model with all four controls and can be found in Section 4 of the Supplementary Material.

^7^https://coronavirus.jhu.edu/region/japan, last accessed Jan 27, 2022.

^8^See in particular the data collected at: https://projekte.uni-erfurt.de/cosmo2020/web/topic/wissen-verhalten/30-schutzmassnahmen/#verhalten-im-zeitverlauf, last accessed Jan 27, 2022 and https://coronavirus.jhu.edu/region/germany, last accessed Jan 27, 2022.
