# Supplementary material for: Social Norms and Preventive Behaviors in Japan and Germany During the COVID-19 Pandemic
Source: Front Public Health. 2022 Apr 1;10:842177. doi: 10.3389/fpubh.2022.842177 (PMC9010522; doi:10.3389/fpubh.2022.842177)
Supplement: Supplementary file 1 [file Data_Sheet_1.PDF]

Supplementary Material

S1: Variable Definitions

Table S1. Definitions of dependent variables

Variable	Definition	Year	
		Germany	*Japan*
Preventive attitudes			
Willingness to get vaccinated if vaccine is safe and effective	Indicator variable:<br>1: yes, 0: no	June-July 2020	Oct-Nov 2020
Mandatory vaccination policy	Indicator variables:<br>1: support, 0: no support	June-July 2020	Oct-Nov 2020
Preventive behaviors			
Avoided contact with the elderly and chronically ill.	Indicator variable:<br>1: yes, 0: no	April-July 2020	Oct-Nov 2020
Avoided using public transportation.	Indicator variable:<br>1: yes, 0: no	April-July 2020	Oct-Nov 2020
Refrained from travel, including domestic travel.	Indicator variable:<br>1: yes, 0: no	April-July 2020	Oct-Nov 2020
Avoided peak-hour shopping.	Indicator variable:<br>1: yes, 0: no	April-July 2020	Oct-Nov 2020
Avoiding closed spaces, crowded places, and close-contact settings ("three Cs").	Indicator variable:<br>1: yes, 0: no	April-July 2020	Oct-Nov 2020
Kept a distance from people with coughs, colds, or fevers.	Indicator variable:<br>1: yes, 0: no	April-July 2020	Oct-Nov 2020
Avoided physical contact, such as shaking hands.	Indicator variable:<br>1: yes, 0: no	April-July 2020	Oct-Nov 2020
I washed my hands regularly.	Indicator variable:<br>1: yes, 0: no	April-July 2020	Oct-Nov 2020

Note: In Germany, respondents were asked whether they engaged in the preventive behaviors in the seven days prior to the interview. In Japan, respondents were asked whether they engaged in the preventive behaviors in September.

Table S1. Definitions of independent variables

Variable	Definition	Year	
		Germany	*Japan*
Sociodemographic			
Female	Indicator variable:<br>1: female respondents, 0: else	2019 or 2018	2019
Age	Age in years	2018	2019
Tertiary education	Indicator variable:<br>1: respondents with tertiary education (SOEP: categories 8 and 9 in CASMIN-Scale; KHPS: codes 4 (university) and 5 (grad school))<br>0: else	2018 or 2017	2019
Net monthly household income	Self-reported value in k USD 2019 PPP	2019 or 2018	2019
Children below age 17	Indicator variable:<br>1: at least one child below age 16 living in household<br>0: else	2019	2019
Health			
Health status	Self-assessed health status, measured on 5-point Likert scale: 1 "very good"; 5: "bad"<br>Standardized to mean zero and SD 1	2019 or 2018	2019
Number of risky diseases	Number of diseases associated with complicated COVID-19 infection and available in KHPS and SOEP: diabetes, heart disease, high blood pressure and obesity	2019	2019
Big Five			
Extraversion	Averages based on the respective items from the BIG-5 item batteries<br>Measured on a 7-point Likert scale and standardized to mean zero and SD 1	2019	2019
Conscientiousness		2019	2019
Openness to experience		2019	2019
Neuroticism		2019	2019
Agreeableness		2019	2019
Regional (D: NUTS2, J: prefecture)			
COVID-19 cases	Sum of COVID-19 cases per 100k inhabitants in the region in last 14 days before the day of interview	2020	2020
Weekly change of COVID-19 cases	Weekly change of COVID-19 cases in %	2020	2020
Nominal GDP per capita	Nominal GDP of the region in thousand USD 2019 PPP	2018	2017
Population density	Population density of the region in people / square km	2019	2017
Unemployment rate	Unemployment rate of the region in %	2019	2019

Construction of Big 5 personality traits

In SOEP, each dimension of the Big Five is surveyed using three to four items. In KHPS, each dimension is surveyed using two to three items. Items with negative polarity (-) are recoded so that they have a positive polarity. This means that higher levels of this item are associated with a higher level of the underlying personality trait.

Table S2: Big Five items in SOEP and KHPS

Big Five	Items in SOEP	Items in KHPS
Extraversion	I am outgoing, sociable	I am active and extraverted
	I am reserved (-)	I am reserved and gentle (-)
	I am communicative, talkative	
Conscientiousness	I am somewhat lazy (-)	I am lazy (-)
	I am effective and efficient in completing tasks	I can manage by myself and am strict to myself
	I am a thorough worker	
Openness to experience	I am original and bring in new ideas	I am an ordinary person without new ideas (-)
	I am imaginative	I like new things and I tend to have different ideas compared to others
	I am eager for knowledge	I am interested in new things every year
	I appreciate artistic and aesthetic experiences	
Neuroticism	I am often worried	I think I'm a worrier and easily flustered
	I am relaxed, able to deal with stress (-)	I am relaxed and handle stress (-)
	I am nervous	
Agreeableness	I am sometimes a bit rude to others (-)	I am likely to be dissatisfied with others and to cause conflicts (-)
	I am considerate and kind to others	I care about others and I am a kind person
	I am forgiving	

S2: Data Sources

Table S4. Sources of regional data

Variable	Data source (Germany)	Data source (Japan)
(Weekly change of) COVID-19 cases	Robert-Koch-Institut npgeo-corona-npgeo-de.hub.arcgis.com/datasets/dd4580c810204019a7b8eb3e0b329dd6_0	Nippon Hōsō Kyōkai www3.nhk.or.jp/news/special/coronavirus/data/
Nominal GDP per capita	Federal Statistical Office statistikportal.de/de/vgrdl/ergebnisse-kreisebene/bruttoinlandsprodukt-bruttowertschoepfung-kreise	Prefectural Accounts, Cabinet Office, Japan www.esri.cao.go.jp/jp/sna/data/data_list/kenmin/files/files_kenmin.html
Population density	Federal Statistical Office destatis.de/DE/Themen/Laender-Regionen/Regionales/Gemeindeverzeichnis/Administrativ/04-kreise.html	Population Census (for population) and Japan Statistical Year Book (for area), Ministry of Internal Affairs and Communications, Japan
Unemployment rate	Federal Employment Agency statistik.arbeitsagentur.de/SiteGlobals/Forms/Suche/Einzelheftsuche_Formular.html?submit=Suchen&topic_f=gemeinde-arbeitslose-quoten	Labor Force Survey, Ministry of Internal Affairs and Communications, Japan

S3: Description of Blinder-Oaxaca Decomposition Method

The Blinder-Oaxaca decomposition is a method to decompose the mean differences in the outcomes into a part that is due to differences in the mean values of the explanatory variables and a part that is due to differences in the correlations of the dependent variables with the explanatory variable (1; 2). While there are different subtypes of Blinder-Oaxaca decompositions (3), we focus on the decomposition originally proposed by Blinder (1). It allows us to decompose the raw average difference in outcomes into two parts: an explained part, i.e., mean outcome differences that can be explained by mean difference in covariates, and an unexplained part, i.e., mean outcome differences arising from differing coefficients. Following Yun (4), one can derive the decomposition for a general case of a possibly non-linear relationship between the covariates and the outcome.

Assume that the $Nx1$ vector of outcomes y_j for groups $j = D, J$ depends on a linear combination of the covariates via the possibly non-linear, once-differentiable function F, i.e.,

$$y_j = F(X_j \beta_j)$$

where X_j is a NxK matrix of covariates and β_j is a $Kx1$ vector of coefficients. Then the raw difference in average outcomes R given by

$$\mathrm{R} = \overline{\mathrm{y_D}} - \overline{\mathrm{y_J}}$$

which can be rewritten as

$$\mathrm{R} = \overline{\mathrm{F}(X_D \beta_D)} - \overline{\mathrm{F}(X_J \beta_J)}.$$

Bars indicate averages. Adding and subtracting the average, counterfactual outcome of an individual from J if she had the average coefficient vector from D, $\overline{\mathrm{F}(X_J \beta_D)}$, we obtain the decomposition

$$\mathrm{R} = \underbrace{\overline{\mathrm{F}(X_D \beta_D)} - \overline{\mathrm{F}(X_J \beta_D)}}_{\text{Explained difference (E)}} + \underbrace{\overline{\mathrm{F}(X_J \beta_D)} - \overline{\mathrm{F}(X_J \beta_J)}}_{\text{Unexplained difference (U)}} = \mathrm{E} + \mathrm{U}.$$

We limit our attention to the explained part of the difference. This explained part can be further decomposed into the contribution of individual covariates. This is known in the literature as detailed decomposition as opposed to the aggregate decomposition. If F is a linear function, the detailed decomposition is straightforward to derive. If F is non-linear, as it is in our case given that we estimate a logit model, Yun (2004) shows that the explained difference can be decomposed into the contribution of differences in the K covariates by using appropriate weights estimated from the data. In particular,

$$\mathrm{E} = \sum_{\mathrm{k}=1}^{\mathrm{K}} \mathrm{E_k} = \sum_{\mathrm{k}=1}^{\mathrm{K}} \mathrm{W}_{\Delta \mathrm{X}}^{\mathrm{k}} \left[\overline{\mathrm{F}(\mathrm{X_D}\beta_\mathrm{D})} - \overline{\mathrm{F}(\mathrm{X_J}\beta_\mathrm{D})}\right] + \sum_{k=1}^{K} W_{\Delta\beta}^{k} \left[\overline{\mathrm{F}(X_J \beta_D)} - \overline{\mathrm{F}(X_J \beta_J)}\right],$$

where

$$W_{\Delta \mathrm{X}}^{k} = \frac{\left(\overline{\mathrm{X_D^k}} - \overline{\mathrm{X_J^k}}\right)\beta_\mathrm{D}^\mathrm{k}}{\left(\overline{\mathrm{X_D}} - \overline{\mathrm{X_J}}\right)\beta_\mathrm{D}},$$

$$W_{\Delta\beta}^{k} = \frac{\overline{\mathrm{X_J^k}}\left(\beta_\mathrm{D}^\mathrm{k} - \beta_\mathrm{J}^\mathrm{k}\right)}{\overline{\mathrm{X_J}}\left(\beta_\mathrm{D} - \beta_\mathrm{J}\right)} \text{ and}$$

$$\sum_{k=1}^{K} W_{\Delta \mathrm{X}}^{k} = \sum_{k=1}^{K} W_{\Delta\beta}^{k} = 1.$$

Using the *oaxaca* command in Stata (3), we use this generalised Blinder-Oaxaca decomposition based on estimating a logit model.

S4: Supplementary estimation results

To analyze the influence of the covariates on preventive attitudes and activities, we estimated five different regression specifications for each of the ten dependent variables: The first four specifications each used a single set of the total four sets of covariates, the fifth included all four sets simultaneously. Having shown the results for the fifth specification in the main body of the text, we now present the regression coefficients for the first four specifications as a robustness check. For each dependent variable, we compare these specifications to a case where the dependent variable is only regressed on the country dummy.

Note for Tables S5 to S14:
Data on the individual level from SOEP-CoV and KHPS-CoV. For regional data sources, please refer to Table S2 of this Supplement. All numbers are weighted. Regression coefficients. Coefficient for the intercept is not shown. The Big Five and self-assessed health are measured in standard deviations. Section 1 of this Appendix provides definitions of all the variables and details on the construction of the Big Five. Rows with a (D) give the effect for the German sample. Rows with a Δ give the differential effect for the Japanese sample. * $p<0.1$, ** $p<0.05$, *** $p<0.01$.

Table S5. Additional specifications for willingness to get vaccinated

**Covariates**	**Country dummy**	**Sociodemo-graphic**	**Health**	**Big Five**	**Regional**
Japan	0.929***	3.431***	0.979***	0.914***	0.914
Sociodemographic					
Female (D)		-0.867***			
Δ Female (J)		0.714**			
Age (D)		0.026***			
Δ Age (J)		-0.034***			
Tertiary education (D)		1.377***			
Δ Tertiary education (J)		-1.310***			
Net monthly household income (D)		0.128**			
Δ Net monthly household income (J)		-0.121*			
Share with children of age<17 (D)		0.432			
Δ Share with children of age<17 (J)		-0.656			
Health					
Self-assessed health (D)			-0.029		
Δ Self-assessed health (J)			0.138		
Number of risky diseases (D)			0.118		
Δ Number of risky diseases (J)			0.001		
Big Five					
Extraversion (D)				-0.396***	
Δ Extraversion (J)				0.391**	
Conscientiousness (D)				-0.114	
Δ Conscientiousness (J)				0.134	
Openness to experience (D)				0.382***	
Δ Openness to experience (J)				-0.387***	
Neuroticism (D)				-0.161	
Δ Neuroticism (J)				-0.065	
Agreeableness (D)				0.098	
Δ Agreeableness (J)				-0.121	
Regional (D: NUTS2, J: prefecture)					
COVID-19 cases in last 14 days (D)					0.043**
Δ COVID-19 cases in last 14 days (J)					-0.082***
Weekly change COVID-19 cases (D)					-0.339
Δ Weekly change COVID-19 cases (J)					0.373
Nominal GDP per capita (D)					0.005
Δ Nominal GDP per capita (J)					0.019
Population density (D)					-0.229
Δ Population density (J)					0.195
Unemployment rate (D)					0.088
Δ Unemployment rate (J)					0.001
**Sample size**	**3,297**	**2,893**	**3,236**	**3,246**	**3,179**

Table S6. Additional specifications for mandatory vaccination

**Covariates**	**Country dummy**	**Sociodemo-graphic**	**Health**	**Big Five**	**Regional**
Japan	-0.170	1.356**	-0.044	-0.152	-0.121
Sociodemographic					
Female (D)		-0.261			
Δ Female (J)		-0.052			
Age (D)		0.030***			
Δ Age (J)		-0.027***			
Tertiary education (D)		0.133			
Δ Tertiary education (J)		-0.301			
Net monthly household income (D)		-0.040			
Δ Net monthly household income (J)		0.054			
Share with children of age<17 (D)		0.490			
Δ Share with children of age<17 (J)		-0.495			
Health					
Self-assessed health (D)			0.012		
Δ Self-assessed health (J)			-0.004		
Number of risky diseases (D)			0.400***		
Δ Number of risky diseases (J)			-0.243		
Big Five					
Extraversion (D)				-0.060	
Δ Extraversion (J)				0.041	
Conscientiousness (D)				0.077	
Δ Conscientiousness (J)				-0.144	
Openness to experience (D)				0.032	
Δ Openness to experience (J)				-0.096	
Neuroticism (D)				-0.295**	
Δ Neuroticism (J)				0.175	
Agreeableness (D)				0.041	
Δ Agreeableness (J)				-0.084	
Regional (D: NUTS2, J: prefecture)					
COVID-19 cases in last 14 days (D)					-0.015
Δ COVID-19 cases in last 14 days (J)					0.016
Weekly change COVID-19 cases (D)					0.209
Δ Weekly change COVID-19 cases (J)					-0.196
Nominal GDP per capita (D)					-0.000
Δ Nominal GDP per capita (J)					0.008
Population density (D)					-0.208
Δ Population density (J)					0.147
Unemployment rate (D)					0.159*
Δ Unemployment rate (J)					-0.036
**Sample size**	**3,896**	**3,403**	**3,832**	**3,840**	**3,755**

Table S7. Additional specifications for avoiding contact with the elderly

**Covariates**	**Country dummy**	**Sociodemo-graphic**	**Health**	**Big Five**	**Regional**
Japan	-0.726***	-0.682*	-0.650***	-0.697***	0.025
Sociodemographic					
Female (D)		0.046			
Δ Female (J)		0.120			
Age (D)		-0.004			
Δ Age (J)		-0.000			
Tertiary education (D)		0.021			
Δ Tertiary education (J)		0.276*			
Net monthly household income (D)		0.048*			
Δ Net monthly household income (J)		-0.061**			
Share with children of age<17 (D)		-0.103			
Δ Share with children of age<17 (J)		0.228			
Health					
Self-assessed health (D)			-0.077		
Δ Self-assessed health (J)			0.019		
Number of risky diseases (D)			0.025		
Δ Number of risky diseases (J)			-0.128		
Big Five					
Extraversion (D)				-0.023	
Δ Extraversion (J)				0.047	
Conscientiousness (D)				0.051	
Δ Conscientiousness (J)				-0.160**	
Openness to experience (D)				0.028	
Δ Openness to experience (J)				-0.054	
Neuroticism (D)				-0.050	
Δ Neuroticism (J)				-0.019	
Agreeableness (D)				0.085*	
Δ Agreeableness (J)				-0.055	
Regional (D: NUTS2, J: prefecture)					
COVID-19 cases in last 14 days (D)					0.009***
Δ COVID-19 cases in last 14 days (J)					0.008
Weekly change COVID-19 cases (D)					-0.434***
Δ Weekly change COVID-19 cases (J)					0.405***
Nominal GDP per capita (D)					0.002
Δ Nominal GDP per capita (J)					-0.004
Population density (D)					-0.094
Δ Population density (J)					0.138*
Unemployment rate (D)					0.058
Δ Unemployment rate (J)					-0.134
**Sample size**	**9,725**	**8,803**	**9,231**	**9,353**	**9,585**

Table S8. Additional specifications for avoiding public transport

**Covariates**	**Country dummy**	**Sociodemo-graphic**	**Health**	**Big Five**	**Regional**
Japan	-0.714***	-0.893**	-0.643***	-0.704***	-0.753
Sociodemographic					
Female (D)		0.188*			
Δ Female (J)		0.045			
Age (D)		-0.003			
Δ Age (J)		0.013**			
Tertiary education (D)		-0.316**			
Δ Tertiary education (J)		-0.155			
Net monthly household income (D)		0.105**			
Δ Net monthly household income (J)		-0.136***			
Share with children of age<17 (D)		0.006			
Δ Share with children of age<17 (J)		0.434**			
Health					
Self-assessed health (D)			-0.107**		
Δ Self-assessed health (J)			0.161**		
Number of risky diseases (D)			0.052		
Δ Number of risky diseases (J)			-0.117		
Big Five					
Extraversion (D)				0.043	
Δ Extraversion (J)				-0.149*	
Conscientiousness (D)				0.199***	
Δ Conscientiousness (J)				-0.270***	
Openness to experience (D)				-0.056	
Δ Openness to experience (J)				0.201**	
Neuroticism (D)				0.109*	
Δ Neuroticism (J)				-0.211***	
Agreeableness (D)				0.009	
Δ Agreeableness (J)				-0.041	
Regional (D: NUTS2, J: prefecture)					
COVID-19 cases in last 14 days (D)					0.009***
Δ COVID-19 cases in last 14 days (J)					-0.027*
Weekly change COVID-19 cases (D)					-0.207
Δ Weekly change COVID-19 cases (J)					0.197
Nominal GDP per capita (D)					-0.004
Δ Nominal GDP per capita (J)					0.018**
Population density (D)					-0.203***
Δ Population density (J)					-0.004
Unemployment rate (D)					0.049
Δ Unemployment rate (J)					0.025
**Sample size**	**9,732**	**8,813**	**9,238**	**9,360**	**9,592**

Table S9. Additional specifications for avoiding travel

**Covariates**	**Country dummy**	**Sociodemo-graphic**	**Health**	**Big Five**	**Regional**
Japan	-0.086	-0.731	-0.015	-0.050	-0.109
Sociodemographic					
Female (D)		0.422***			
Δ Female (J)		0.109			
Age (D)		-0.003			
Δ Age (J)		0.017**			
Tertiary education (D)		-0.397***			
Δ Tertiary education (J)		-0.080			
Net monthly household income (D)		0.020			
Δ Net monthly household income (J)		-0.066*			
Share with children of age<17 (D)		0.122			
Δ Share with children of age<17 (J)		-0.063			
Health					
Self-assessed health (D)			-0.045		
Δ Self-assessed health (J)			0.223**		
Number of risky diseases (D)			0.019		
Δ Number of risky diseases (J)			-0.129		
Big Five					
Extraversion (D)				0.158**	
Δ Extraversion (J)				-0.084	
Conscientiousness (D)				-0.009	
Δ Conscientiousness (J)				-0.080	
Openness to experience (D)				-0.005	
Δ Openness to experience (J)				0.182	
Neuroticism (D)				0.157**	
Δ Neuroticism (J)				-0.269**	
Agreeableness (D)				0.039	
Δ Agreeableness (J)				-0.196*	
Regional (D: NUTS2, J: prefecture)					
COVID-19 cases in last 14 days (D)					0.013***
Δ COVID-19 cases in last 14 days (J)					-0.004
Weekly change COVID-19 cases (D)					-0.395***
Δ Weekly change COVID-19 cases (J)					0.422***
Nominal GDP per capita (D)					-0.006
Δ Nominal GDP per capita (J)					0.018
Population density (D)					0.024
Δ Population density (J)					-0.156
Unemployment rate (D)					0.061
Δ Unemployment rate (J)					-0.000
**Sample size**	**9,730**	**8,811**	**9,236**	**9,358**	**9,589**

Table S10. Additional specifications for avoiding peak-hour shopping

**Covariates**	**Country dummy**	**Sociodemo-graphic**	**Health**	**Big Five**	**Regional**
Japan	-0.448***	0.743**	-0.307***	-0.454***	-0.682
Sociodemographic					
Female (D)		0.475***			
Δ Female (J)		-0.227			
Age (D)		0.012***			
Δ Age (J)		-0.016***			
Tertiary education (D)		-0.043			
Δ Tertiary education (J)		0.107			
Net monthly household income (D)		0.035			
Δ Net monthly household income (J)		-0.074**			
Share with children of age<17 (D)		-0.130			
Δ Share with children of age<17 (J)		-0.021			
Health					
Self-assessed health (D)			-0.150***		
Δ Self-assessed health (J)			0.184**		
Number of risky diseases (D)			0.290***		
Δ Number of risky diseases (J)			-0.334***		
Big Five					
Extraversion (D)				-0.074	
Δ Extraversion (J)				0.116	
Conscientiousness (D)				0.161***	
Δ Conscientiousness (J)				-0.245***	
Openness to experience (D)				0.111*	
Δ Openness to experience (J)				-0.137*	
Neuroticism (D)				0.097*	
Δ Neuroticism (J)				-0.147*	
Agreeableness (D)				0.038	
Δ Agreeableness (J)				-0.037	
Regional (D: NUTS2, J: prefecture)					
COVID-19 cases in last 14 days (D)					0.008***
Δ COVID-19 cases in last 14 days (J)					0.002
Weekly change COVID-19 cases (D)					-0.161
Δ Weekly change COVID-19 cases (J)					0.161
Nominal GDP per capita (D)					-0.003
Δ Nominal GDP per capita (J)					0.006
Population density (D)					-0.164**
Δ Population density (J)					0.170**
Unemployment rate (D)					0.014
Δ Unemployment rate (J)					0.038
**Sample size**	**9,734**	**8,812**	**9,240**	**9,362**	**9,593**

Table S11. Additional specifications for avoiding crowds

**Covariates**	**Country dummy**	**Sociodemo-graphic**	**Health**	**Big Five**	**Regional**
Japan	-0.545***	-1.069*	-0.465***	-0.562***	-0.196
Sociodemographic					
Female (D)		0.491***			
Δ Female (J)		0.251			
Age (D)		0.002			
Δ Age (J)		0.004			
Tertiary education (D)		-0.187			
Δ Tertiary education (J)		0.645**			
Net monthly household income (D)		-0.012			
Δ Net monthly household income (J)		-0.007			
Share with children of age<17 (D)		-0.038			
Δ Share with children of age<17 (J)		0.108			
Health					
Self-assessed health (D)			0.009		
Δ Self-assessed health (J)			-0.153		
Number of risky diseases (D)			0.232		
Δ Number of risky diseases (J)			-0.279		
Big Five					
Extraversion (D)				0.046	
Δ Extraversion (J)				-0.010	
Conscientiousness (D)				0.084	
Δ Conscientiousness (J)				-0.322**	
Openness to experience (D)				0.009	
Δ Openness to experience (J)				0.112	
Neuroticism (D)				0.301***	
Δ Neuroticism (J)				-0.299**	
Agreeableness (D)				0.091	
Δ Agreeableness (J)				-0.045	
Regional (D: NUTS2, J: prefecture)					
COVID-19 cases in last 14 days (D)					0.012***
Δ COVID-19 cases in last 14 days (J)					-0.009
Weekly change COVID-19 cases (D)					-0.367
Δ Weekly change COVID-19 cases (J)					0.361
Nominal GDP per capita (D)					-0.006
Δ Nominal GDP per capita (J)					0.014
Population density (D)					-0.129
Δ Population density (J)					0.166
Unemployment rate (D)					0.069
Δ Unemployment rate (J)					-0.261
**Sample size**	**9,740**	**8,817**	**9,246**	**9,368**	**9,599**

Table S12. Additional specifications for avoiding people with symptoms of a cold

**Covariates**	**Country dummy**	**Sociodemo-graphic**	**Health**	**Big Five**	**Regional**
Japan	0.096	-0.302	0.115	0.052	0.520
Sociodemographic					
Female (D)		0.167			
Δ Female (J)		0.517**			
Age (D)		-0.015***			
Δ Age (J)		0.003			
Tertiary education (D)		0.164			
Δ Tertiary education (J)		-0.084			
Net monthly household income (D)		0.018			
Δ Net monthly household income (J)		-0.041			
Share with children of age<17 (D)		-0.533***			
Δ Share with children of age<17 (J)		0.693**			
Health					
Self-assessed health (D)			-0.071		
Δ Self-assessed health (J)			-0.046		
Number of risky diseases (D)			0.139		
Δ Number of risky diseases (J)			-0.031		
Big Five					
Extraversion (D)				0.002	
Δ Extraversion (J)				-0.056	
Conscientiousness (D)				0.163**	
Δ Conscientiousness (J)				-0.261**	
Openness to experience (D)				-0.064	
Δ Openness to experience (J)				0.137	
Neuroticism (D)				0.165**	
Δ Neuroticism (J)				-0.264**	
Agreeableness (D)				0.160**	
Δ Agreeableness (J)				-0.192*	
Regional (D: NUTS2, J: prefecture)					
COVID-19 cases in last 14 days (D)					0.005**
Δ COVID-19 cases in last 14 days (J)					0.036
Weekly change COVID-19 cases (D)					-0.224
Δ Weekly change COVID-19 cases (J)					0.184
Nominal GDP per capita (D)					-0.002
Δ Nominal GDP per capita (J)					0.008
Population density (D)					-0.118
Δ Population density (J)					0.079
Unemployment rate (D)					0.058
Δ Unemployment rate (J)					-0.296
**Sample size**	**9,727**	**8,808**	**9,233**	**9,355**	**9,586**

Table S13. Additional specifications for avoiding physical contact

**Covariates**	**Country dummy**	**Sociodemo-graphic**	**Health**	**Big Five**	**Regional**
Japan	-0.349***	-0.198	-0.252	-0.358***	0.535
Sociodemographic					
Female (D)		0.123			
Δ Female (J)		0.550**			
Age (D)		0.016***			
Δ Age (J)		-0.003			
Tertiary education (D)		0.089			
Δ Tertiary education (J)		0.217			
Net monthly household income (D)		0.128**			
Δ Net monthly household income (J)		-0.091			
Share with children of age<17 (D)		-0.033			
Δ Share with children of age<17 (J)		0.383			
Health					
Self-assessed health (D)			-0.024		
Δ Self-assessed health (J)			0.131		
Number of risky diseases (D)			0.214		
Δ Number of risky diseases (J)			-0.156		
Big Five					
Extraversion (D)				-0.309***	
Δ Extraversion (J)				0.326**	
Conscientiousness (D)				0.219**	
Δ Conscientiousness (J)				-0.431***	
Openness to experience (D)				0.008	
Δ Openness to experience (J)				0.106	
Neuroticism (D)				0.159*	
Δ Neuroticism (J)				-0.330**	
Agreeableness (D)				0.108	
Δ Agreeableness (J)				-0.124	
Regional (D: NUTS2, J: prefecture)					
COVID-19 cases in last 14 days (D)					0.013***
Δ COVID-19 cases in last 14 days (J)					0.025
Weekly change COVID-19 cases (D)					-0.473**
Δ Weekly change COVID-19 cases (J)					0.440**
Nominal GDP per capita (D)					0.008
Δ Nominal GDP per capita (J)					-0.009
Population density (D)					-0.176
Δ Population density (J)					0.151
Unemployment rate (D)					0.109
Δ Unemployment rate (J)					0.026
**Sample size**	**9,737**	**8,815**	**9,243**	**9,365**	**9,596**

Table S14. Additional specifications for washing hands regularly

**Covariates**	**Country dummy**	**Sociodemo-graphic**	**Health**	**Big Five**	**Regional**
Japan	-1.550***	-0.222	-1.575***	-1.758***	-1.210
Sociodemographic					
Female (D)		0.740***			
Δ Female (J)		-0.047			
Age (D)		0.005			
Δ Age (J)		-0.016			
Tertiary education (D)		0.348			
Δ Tertiary education (J)		-0.365			
Net monthly household income (D)		0.100*			
Δ Net monthly household income (J)		-0.114*			
Share with children of age<17 (D)		0.057			
Δ Share with children of age<17 (J)		0.154			
Health					
Self-assessed health (D)			0.027		
Δ Self-assessed health (J)			-0.148		
Number of risky diseases (D)			0.033		
Δ Number of risky diseases (J)			-0.048		
Big Five					
Extraversion (D)				0.333***	
Δ Extraversion (J)				-0.383***	
Conscientiousness (D)				0.283*	
Δ Conscientiousness (J)				-0.489***	
Openness to experience (D)				0.009	
Δ Openness to experience (J)				0.031	
Neuroticism (D)				0.109	
Δ Neuroticism (J)				-0.210	
Agreeableness (D)				0.233	
Δ Agreeableness (J)				-0.253	
Regional (D: NUTS2, J: prefecture)					
COVID-19 cases in last 14 days (D)					0.001
Δ COVID-19 cases in last 14 days (J)					0.072***
Weekly change COVID-19 cases (D)					0.052
Δ Weekly change COVID-19 cases (J)					-0.086
Nominal GDP per capita (D)					0.003
Δ Nominal GDP per capita (J)					-0.009
Population density (D)					-0.203
Δ Population density (J)					0.140
Unemployment rate (D)					0.124
Δ Unemployment rate (J)					0.005
**Sample size**	**9,735**	**8,814**	**9,241**	**9,363**	**9,594**

S5: Supplementary References

(1) Oaxaca R. Male-female wage differentials in urban labor markets. *Int Econ Rev* (1973) **14**(3):693–709.

(2) Blinder AS. Wage Discrimination: Reduced Form and Structural Estimates. *J Hum Resour* (1973) **8**(4):436–455. *doi:10.2307/144855*

(3) Jann B. The Blinder-Oaxaca decomposition for linear regression models. *The Stata Journal* (2008) **8**(4):453–479.

(4) Yun MS. Decomposing differences in the first moment. *Econ Lett* (2004) **82**(2):275-280.
